# Evidence-based speech, language and hearing therapy and the Cochrane Library's systematic reviews

**DOI:** 10.1590/S1516-31802006000200001

**Published:** 2006-03-02

**Authors:** Regina Paolucci El Dib, Álvaro Nagib Atallah

With hundreds of health studies being published every month, we felt there was a need to produce a synthesis of the information that is relevant to our field of knowledge. There is also a need for high-quality articles to act as guidelines for clinical practice.^1^

This is one of the objectives of evidence-based medicine, which is an approach that uses the tools of clinical epidemiology, scientific methodology and computer science to examine the existing information, create research results and generate useful knowledge of health-related issues. Through this, consistent evidence for health-related decision-making can be put forward.^2^

Thus, evidence-based medicine is a link between good scientific research and clinical practice. Through clinical epidemiology, strict methods for study design and the preparation, planning, execution and statistical analysis of scientific projects are obtained. In order to obtain reliable evidence and be able to use evidence-based medicine, health professionals must carry out efficient literature searches and select relevant and methodologically appropriate studies.^2^

Therefore, ideally, the practice of evidence-based medicine should be a systematic process that includes various phases: formulation of a relevant clinical question based on the patients' clinical status, literature search for relevant clinical articles, critical analysis of the validity and applicability of the evidence (scientific proof), and lastly, transfer of the important findings to clinical practice.^2^

Evidence-based health is another term in current use among professional groups that already carry out critical evaluation of the literature, in fields like cardiology, psychiatry, gynecology, physiotherapy and other branches of medicine. Within this context, it is also appropriate and necessary to develop evidence-based speech, language and hearing therapy, with the aim of using the same tools as in evidence-based medicine. Through this, we can explore therapeutic issues that gained approval on the basis of poor data (resulting from the scientific methodology employed).

When we speak of the evidence favoring a type of treatment, we consider its effectiveness, efficiency, efficacy and safety. Its effectiveness is an assessment of how well it works under real-world conditions. It efficacy is how well it works under ideal conditions. Its efficiency is a measurement of how inexpensive and accessible it is, so that users can have recourse to it. And lastly, its safety is an evaluation of whether the intervention is reliable and does not lead to effects that the patient does not desire.^2^

Therefore, a study that is both internally and externally valid must present the characteristics described above. When researching information on interventions or prevention, we seek studies that best fit this mold, which means systematic reviews that offer level 1 evidence for healthcare decision-making.^2^

Systematic reviews are thus a type of secondary study. That is to say, they include primary studies (randomized clinical trials) that are methodologically appropriate and stringent, and potentially bias-free. Their purpose is to bring together and organize similar studies, to evaluate their methodology, and, where possible, include them in a meta-analysis.^2^

Meta-analysis is a statistical technique in which all the data from all the available studies on some topic are combined. The technique is used by researchers to get a maximum of statistical information without worrying about distortions in the results.^2^

In dealing with speech, language and hearing therapy, we also feel there is a need to organize the existing information in this field, and thus provide adequate guidelines for therapeutic methods.

The most sensible way of answering our everyday queries is to seek help from sources of evidence-based medicine. The best of these sources is the Cochrane Library, which maps clinical trials according to their methods and combines them when appropriate, based on meta-analysis methodology. This virtual library is considered one of the best sources of evidence for healthcare decision-making. It assembles eight data banks, including the database of systematic reviews and meta-analyses, abstracts of reviews of effects, Cochrane controlled trials register (CENTRAL), methodology reviews, economic evaluation and health technology evaluation.

A review of the literature conducted in the Cochrane Library's database of systematic reviews (issue 1, 2006) on the practices of speech, language and hearing therapy identified 15 systematic reviews and protocols for the terms "dysphagia", "dysarthria", "speech and language therapy", "aphasia", "voice disorders", "audiology", "audiometry", "hearing loss", "myofunctional therapy" and their respective synonyms. These terms, which are specific to speech, language and hearing therapy, were combined using Boolean operators (and, or, and not) in order to narrow the search.

As mentioned, there are 15 reviews and protocols relating to the field of speech, language and hearing therapy. If we consider that there are 2,180 systematic reviews in this database, which cover various healthcare topics, it is clear that speech, language and hearing therapy has a long road to run if it is to answer 50% of the questions posed in this field. We do, however, already have a few studies presenting level 1 evidence, on which to build clinical practice.

We shall now briefly describe the Cochrane systematic reviews relating to the practices of speech, language and hearing therapy. One of the systematic reviews had the aim of establishing the effects of nutritional supplementation through gastrostomy or jejunostomy, in comparison with oral feeding alone, in children with cerebral palsy and a diagnosed eating disorder. No controlled clinical trials were found that met the inclusion criteria for systematic reviews, so there is thus no good evidence available at the moment that allows us to conclude that enteral or stomach feeding can be either beneficial or harmful, in comparison with a control group. We therefore recommend that well-designed clinical trials should be conducted in order to verify whether nutritional supplementation has any effect as an alternative for children with eating disorders.^3^

It is unclear how dysphagic patients should be treated and fed after a cerebrovascular accident. Bath and Bath (2000) conducted a systematic review that had the objective of analyzing strategies to be used among dysphagic patients, with particular attention devoted to when and how to feed them. Only two studies with small samples were included. Among the implications for clinical practice, percutaneous gastrostomy may increase the nutritional outcome, in comparison with nasogastric tubing in dysphagic patients. However, more research is needed in order to know when and how to feed these patients, and also to analyze the effects of language therapy and medication therapy on dysphagia.^4^

Dysphagia is a common complication in progressive muscle disorders, both in adults and children, and one that is currently not properly understood. Hill et al. (2004) determined the most appropriate intervention for patients with muscle disorders, including consideration of randomized and quasi-randomized clinical trials. No study that met the inclusion criteria was found. It was thus impossible to determine what the best intervention for treating dysphagia was.^5^

Deane et al. (2001) compared the efficacy and effectiveness of non-pharmacological therapy with placebo or no intervention in patients with Parkinson's disease with dysphagia. No controlled clinical trial was found that met the inclusion criteria. There was therefore no evidence that supports the efficacy of this therapy. It was suggested that rigorously planned controlled clinical trials should be conducted.^6^

After searching through the Cochrane systematic reviews, we found one review protocol, i.e. one ongoing systematic review that was designed to compare the efficacy of different interventions in dysphagic patients with esophageal cancer. As this is only a protocol, the authors are still in the process of extracting and analyzing the data.^7^

Aphasia can be defined as language impairment due to a brain lesion. With regard to its treatment, Greener et al. (2000) established that speech and language therapy supplied by a qualified professional was effective, in comparison with informal therapy, i.e. therapy supplied by someone who is not formally trained or qualified in this field. The controlled clinical trials identified were not methodologically robust enough to allow a conclusion regarding whether intervention is more effective in aphasic patients than in control groups.^8^

Likewise, we retrieved a systematic review that evaluated the efficacy of speech and language therapy in adult patients with dysarthria. Dysarthria is the condition of difficulty in speech articulation (abnormalities in the neuromuscular control of palate, tongue and lip movements). Sellars et al. (2005) concluded that there was not enough evidence to answer this question. In other words, there were no methodologically sound studies that could be included in the systematic review. The authors ended by stating that there was a fundamental need for high-quality research in this field.^9^

Deane et al. (2001) reviewed treatments for patients with dysarthria, using Parkinson's disease as the clinical situation, speech and language therapy as the intervention, placebo for the control group, and the ability to communicate with intelligible speech as the clinical outcome. Three randomized clinical trials were included, with a total of 63 patients. Considering the limited sample size, the inadequate methodology of the clinical trials, and the possibility of publication bias, there was insufficient evidence to establish whether language and speech therapy among dysarthric patients is efficacious. The authors suggested that more controlled clinical trials with larger numbers of participants should be conducted.^10^

Deane et al. (2001) also carried out another systematic review with the aim of comparing the use of different speech and language therapy techniques with the same population. They came to the same conclusion as in the previous revision: there was not enough evidence to answer the clinical question.^11^

Law et al. (2003) aimed to determine the effectiveness of speech therapy in children with primary speech and language disability. The results suggested that therapy was effective for children with vocabulary and phonological difficulties. However, the findings were heterogeneous, and it was concluded that more clinical trials were needed to prove the effectiveness of speech and language therapy among children with receptive language disorders.^12^

The production of speech, language and communicative gestures is usually affected by cerebral palsy. Those responsible for children with cerebral palsy make use of speech and language therapy to improve these children's interactions with the world that surrounds them, and if possible make them a little more independent in their environment. Thus, Pennington et al. (2004) analyzed the effectiveness of speech and language therapy among children with cerebral palsy with communication disorders. There was no hard evidence for any positive effects from speech and language therapy on children with cerebral palsy. It was concluded that rigorous research needed to be carried out to confirm the link between speech and language therapy and improvement in communication in these children.^13^

Apraxia is a neuropsychological disorder that can affect patients who have suffered a cardiovascular accident. It can be defined as a disorder that affects how articular movements are performed, because of a lesion in the area responsible for programming, planning and executing the muscle movements needed to produce words. West et al. (2005) assessed whether therapeutic interventions improve functional speech in stroke patients with apraxia of speech, and which individual therapeutic interventions are effective. No trials were identified. There was no evidence from randomized trials to support or refute the effectiveness of therapeutic interventions for apraxia of speech. It was concluded that there was a need for high-quality randomized trials to be undertaken in this field.^14^

We likewise retrieved a systematic review that had the purpose of analyzing the effectiveness of neonatal hearing screening and early treatment programs for hearing loss. All neonates were screened, as well as children and adults with congenital bilateral hearing loss with a threshold of more than 40 dB. The long-term effectiveness of universal newborn hearing screening programs has not been established to date. It was concluded that there was a need for controlled trials and before and after studies to address this issue further.^15^

El Dib et al. (2005) summarized the evidence regarding whether interventions for encouraging the wearing of hearing protection among workers exposed to noise in the workplace were effective. Two studies were found. The authors concluded there was limited evidence available and that this did not show whether tailored interventions were more or less effective than general interventions among such workers, considering that 80% of them already use hearing protection. Long-lasting school-based interventions might increase the use of hearing protection substantially. These results were based on single studies only. It was concluded that better interventions for increasing the use of hearing protection needed to be developed and evaluated in order to increase the prevention of noise induced hearing loss among workers.^16^

Butler et al. (2003) assessed the evidence from randomized controlled trials regarding the effectiveness of screening and treating children with clinically significant otitis media with effusion (OME) during the first four years of their lives, in relation to language and behavioral outcomes. The authors concluded that the randomized trials identified did not show that there was any significant benefit gained from screening the general population of asymptomatic children during their first four years of life for OME, in relation to language development and behavior.^17^

After reviewing the literature on speech, language and hearing therapy in the systematic review database, we also looked at the Cochrane central register of controlled trials (CENTRAL) and located 1,384 clinical trials (out of a total of 435,786) that were potentially related to speech, language and hearing therapy, using the same terms mentioned earlier. These were also cross-checked through Boolean logical operators. These studies need critical appraisal to evaluate their scientific quality before their incorporation into practice.

## CONCLUSIONS

We were able to identify 15 reviews in the Cochrane Library, of which two were protocols and 13 were complete systematic reviews.

It should be noted that, in all the systematic reviews described above, the authors were unable to provide fundamental practical implications that could act as guidelines for speech and language therapy and consequently reduce the therapeutic uncertainty. On the contrary, the authors' findings were not conclusive enough to be included as interventions in clinical practice. In other words, there was not enough evidence available to establish what effect speech and language therapy has in diverse clinical situations. The best evidence found was not good enough to detect a statistically significant difference in favor of such treatment, because of the small number of studies in the field of speech, language and hearing therapy that were carried out in a methodologically adequate fashion, and with acceptable sample size.

However, the authors were able to highlight the implications for research, and suggested that new well-designed and well-planned controlled clinical trials with larger numbers of participants should be conducted.

Because systematic reviews have a commitment towards updating scientific knowledge, authors will be obliged to include such new methodologically acceptable and homogeneous clinical trials in their systematic reviews, as they are reported. It will be thus possible to add them to meta-analyses and, in so doing, to obtain scientific proof that speech and language therapy works, which is essential for the clinical situations discussed in this article, as well as for other communication disorders.

We know how necessary speech and language therapy is for individuals to succeed in honing their communication skills, as well as for acquiring adequate neurovegetative function and developing the phonoarticulatory organs, to mention only two aspects of hearing loss prevention. However, clinical evidence that one intervention is better than another allows us to refine clinical practice, as we seek new alternatives to hasten patients' progress and recovery.

Even when we really know that one type of treatment is effective, we should assess which approach is most effective among the possible interventions. In such cases it is important to develop new clinical trials. Clinical evidence that one intervention is better than another allows us to improve clinical practice and to search for new alternatives for developing and improving patient care.

As mentioned earlier, the Cochrane Library also includes a database of clinical trials on therapeutic and prophylactic interventions. Any professional interested in these may identify many clinical trials relating to evidence-based speech, language and hearing therapy practice, in order to develop new systematic reviews and better clinical trials, thereby implementing the use of science in this very important field.

The amount of scientific information available regarding speech, language and hearing therapy does not support the belief obtained from practical experience that these techniques are useful.

Colleagues who are interested in this subject may obtain further information from the Cochrane Center site at http://www.centrocochranedobrasil.org.br
